# Methyl 2-hy­droxy-4-iodo­benzoate

**DOI:** 10.1107/S2414314624003948

**Published:** 2024-05-17

**Authors:** Marten J. Kimble, Shea D. Myers, Jason B Benedict

**Affiliations:** a 730 Natural Sciences Complex, Buffalo, NY 14260-3000, USA; b 771 Natural Sciences Complex, Buffalo, NY 14260, USA; University of Aberdeen, United Kingdom

**Keywords:** crystal structure, organic, co-former

## Abstract

The structure of the title compound, C_8_H_7_IO_3_, at 90 K has monoclinic (*P*2_1_/*c*) symmetry. The extended structure is layered and displays inter­molecular and intra­molecular hydrogen bonding arising from the same OH group.

## Abstract 2

The title compound forms a sheet structure of dimers exhibiting intra- and inter­molecular hydrogen bonds.

## Graphical abstract

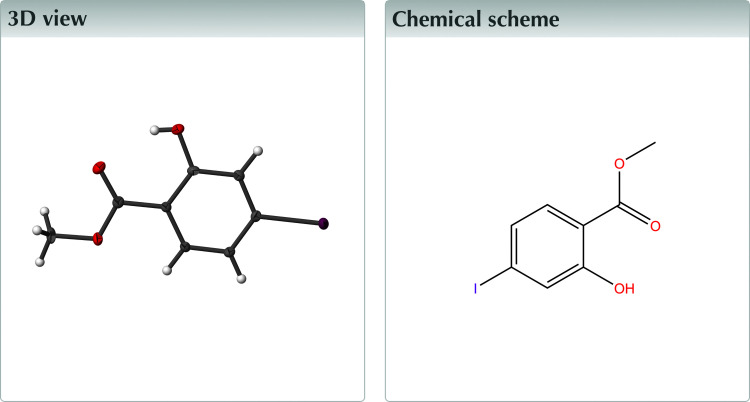

## Structure description

2-Hy­droxy­benzoic acid methyl ester (C_8_H_8_O_3_), commonly known as methyl salicylate, and its derivatives have been shown to display biological effects such as anti-inflammatory, anti-fungal, and process signaling (Yoon *et al.*, 2019 ▸; Li *et al.*, 2016 ▸; Park *et al.*, 2007 ▸). It can also be found in various foods (Duthie & Wood, 2011 ▸). The title compound, 2-hy­droxy-4-iodo­benzoic acid methyl ester (methyl 4-iodo­salicylate, C_8_H_7_IO_3_) allows for an effective way of incorporating the said methyl salicylates within larger organic mol­ecules, using such methodologies as McClure protocols (Franchi *et al.*, 2010 ▸; McClure *et al.*, 2001 ▸), Stille (Yoon *et al.*, 2019 ▸; Stille, 1986 ▸) and Suzuki–Miyaura reactions (Fracaroli *et al.*, 2014 ▸; Miyaura *et al.*, 1979 ▸), which take advantage of the iodine atom at the 4-position of the aromatic ring for the formation of carbon–carbon bonds. The iodine atom is also capable of forming supra­molecular synthons, which may be useful for crystal engineering (Desiraju, 1995 ▸; Cherukuvada *et al.*, 2016 ▸; Mitchell *et al.*, 2023 ▸).

**Figure 1:**
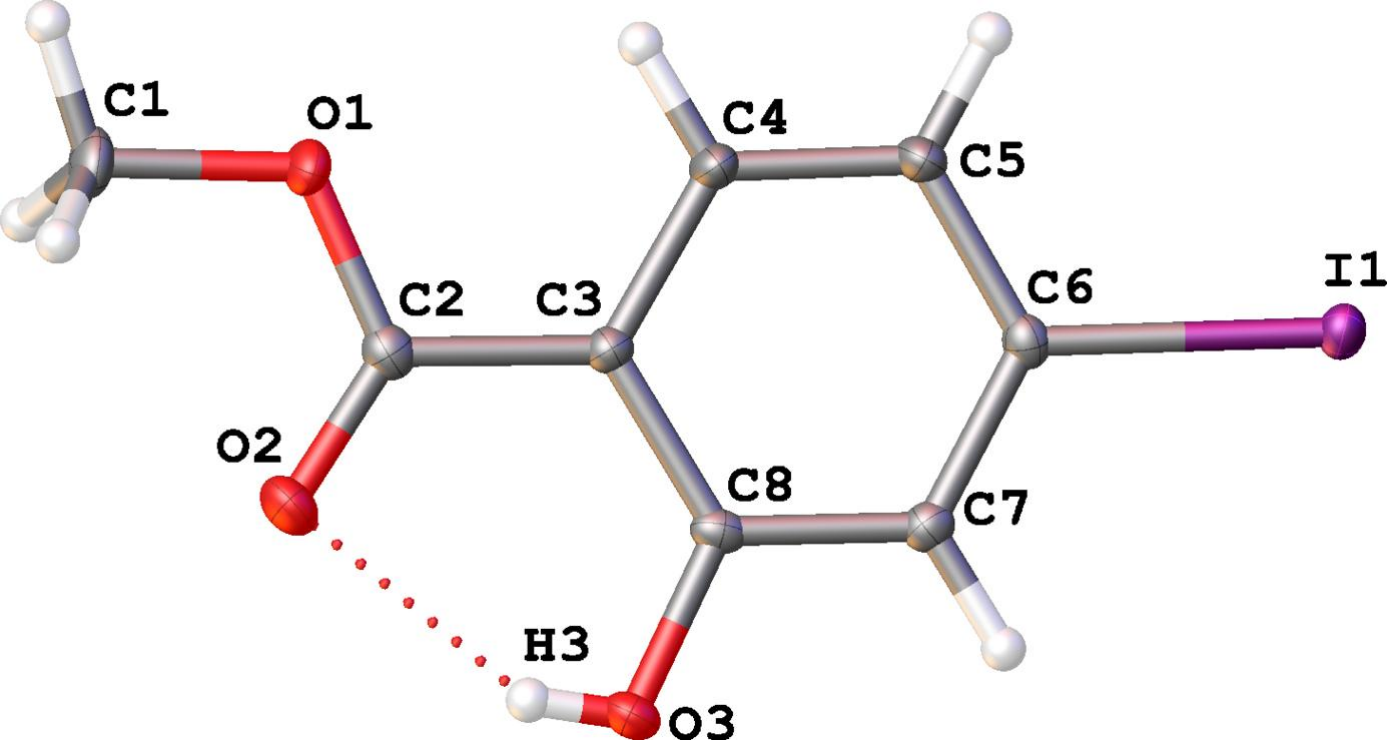
The mol­ecular structure of the title compound showing 50% displacement ellipsoids. The intra­molecular hydrogen bond is indicated by a red dashed line.

**Figure 2:**
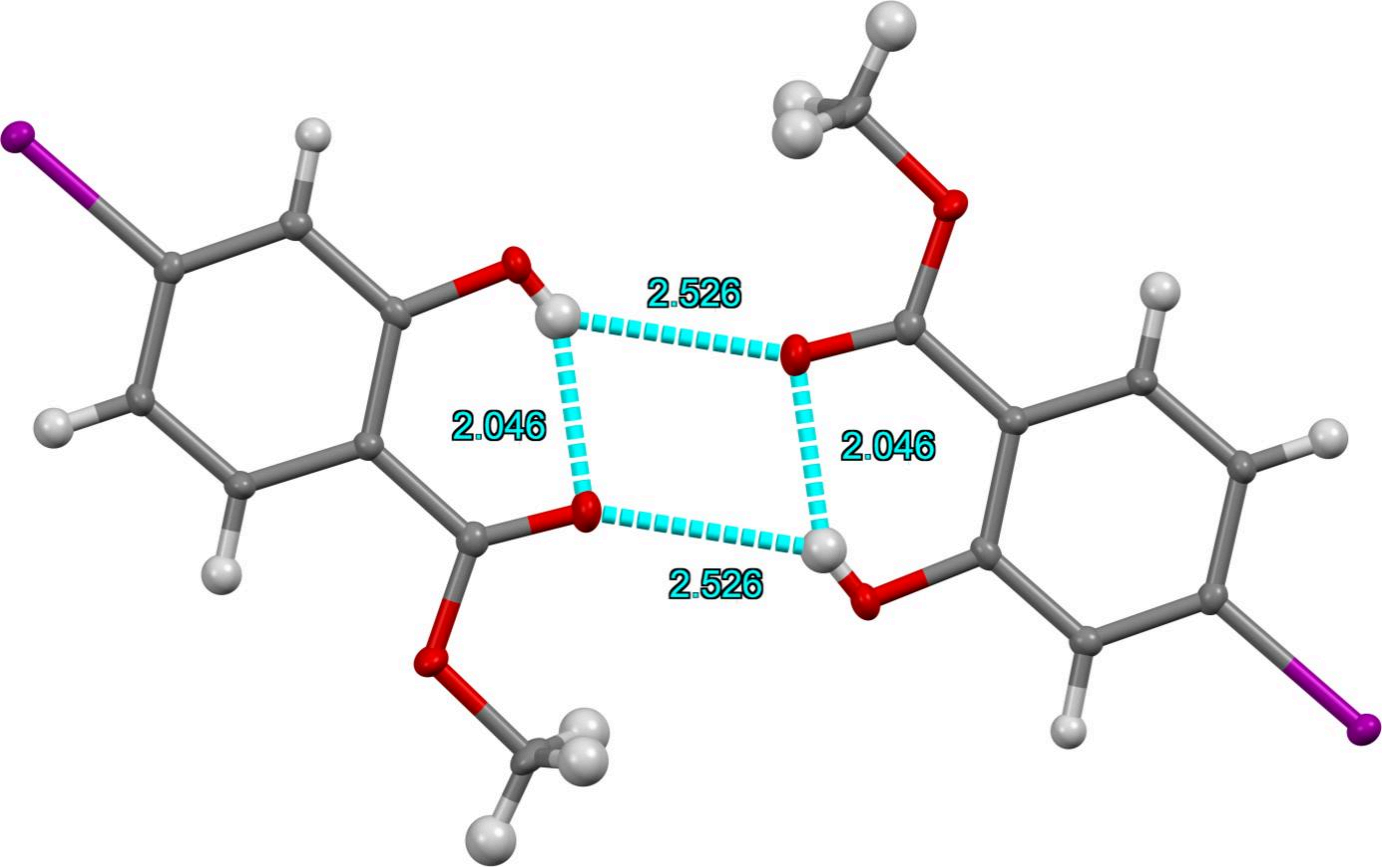
The dimer of title compound showing intra- and inter­molecular hydrogen bonds depicted with blue dashed lines with corresponding O⋯H distances for each O—H⋯O inter­action.

**Figure 3:**
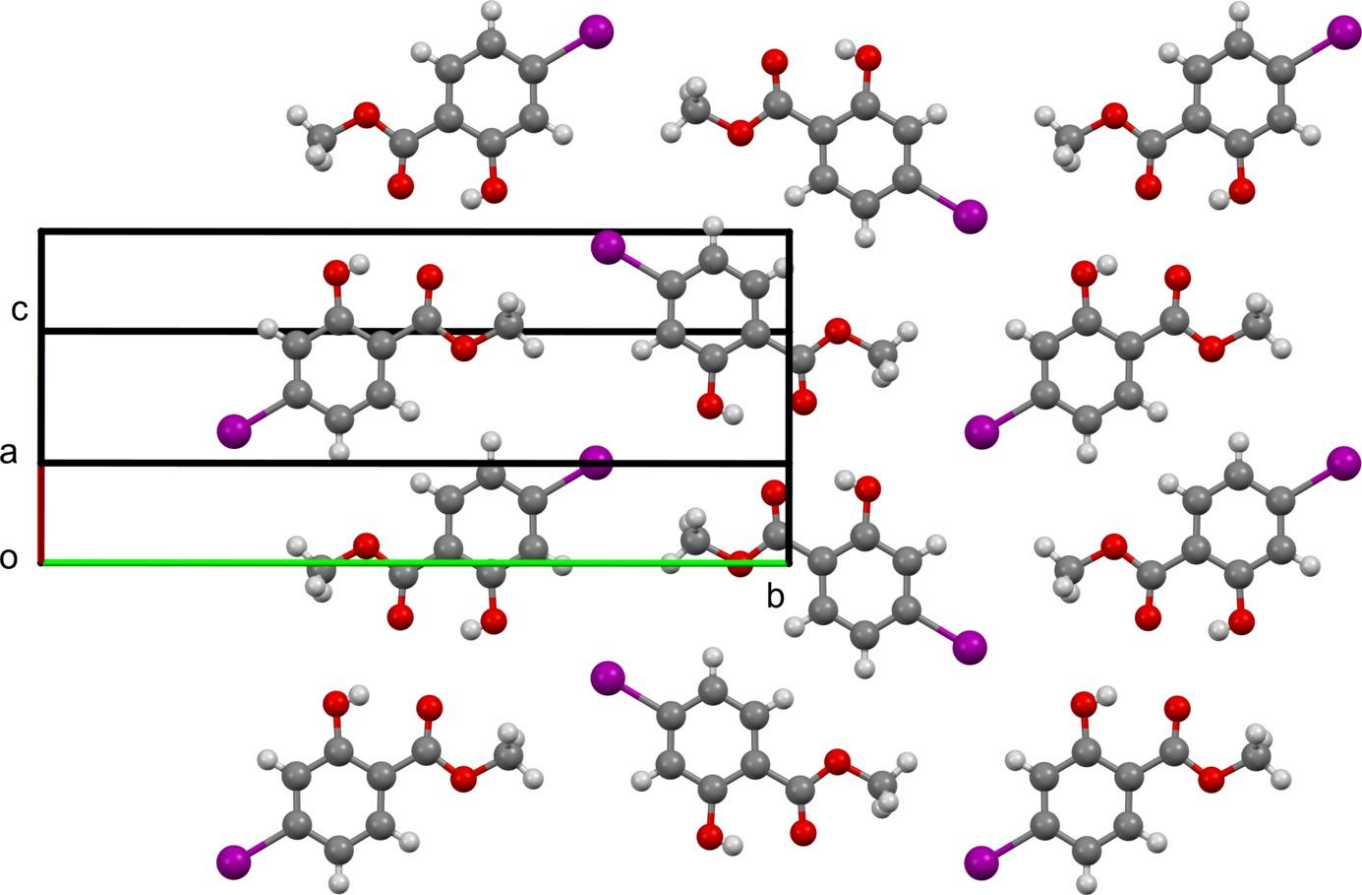
Packing diagram viewed perpendicular to ( 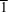 02).

**Figure 4:**
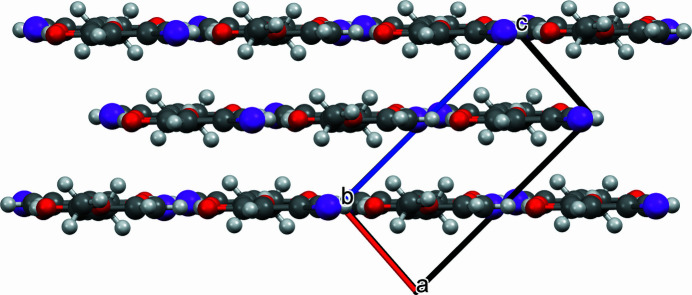
Packing diagram viewed along *b*-axis and parallel to ( 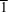 02).

At 90 K the title compound displays monoclinic (*P*2_1_/*c*) symmetry with one mol­ecule in the asymmetric unit (Fig. 1 ▸). Inter­molecular hydrogen bonding inter­actions occur between the hy­droxy groups of one mol­ecule and the carbonyl oxygen atom of the methyl ester of an adjacent mol­ecule to form a centrosymmetric dimeric pair (Table 1 ▸, Fig. 2 ▸) with H⋯O = 2.53 (4) Å. An O3—H3⋯O2 intra­molecular hydrogen bond also exists with an H⋯O distance of 2.05 (4) Å. The C5⋯C8 [3.326 (3) Å] and O3⋯H1*C* (2.51 Å) inter­actions provide the only short contacts between the stacks of offset ( 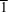 02) parallel sheets, which make up the crystal (Fig. 3 ▸). These sheets, in turn, contain the inversion-generated hydrogen-bonded dimers (Fig. 2 ▸). The non-hydrogen atoms of the mol­ecule are essentially coplanar with no displacement from the mean mol­ecular plane greater than 0.132 Å (Fig. 4 ▸).

## Crystallization

Methyl 4-iodo­salicylate (32.8 mg, 0.118 mmol) was added to a 20 ml scintillation vial to which benzene (∼2 ml) was added, and the vial shaken until the compound dissolved. The resulting solution was then left undisturbed, lightly capped, and in the dark for one week to allow for crystal formation while the solvent slowly evaporated.

## Refinement

Crystal data, data collection, and structure refinement details are summarized in Table 2 ▸.

## Supplementary Material

Crystal structure: contains datablock(s) I. DOI: 10.1107/S2414314624003948/hb4468sup1.cif

Structure factors: contains datablock(s) I. DOI: 10.1107/S2414314624003948/hb4468Isup2.hkl

Supporting information file. DOI: 10.1107/S2414314624003948/hb4468Isup3.cml

CCDC reference: 2352344

Additional supporting information:  crystallographic information; 3D view; checkCIF report

## Figures and Tables

**Table 1:** Hydrogen-bond geometry (Å, °)

*D*—H⋯*A*	*D*—H	H⋯*A*	*D*⋯*A*	*D*—H⋯*A*
O3—H3⋯O2	0.70 (4)	2.05 (4)	2.670 (3)	149 (4)
O3—H3⋯O2^i^	0.70 (4)	2.53 (4)	3.087 (2)	139 (4)

Symmetry code: (i) 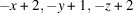 .

**Table 2:** Experimental details

Crystal data	
Chemical formula	C_8_H_7_IO_3_
*M*_r_	278.04
Crystal system, space group	Monoclinic, *P*2_1_/*c*
Temperature (K)	90
*a*, *b*, *c* (Å)	4.3286 (8), 21.334 (4), 9.2941 (16)
β (°)	93.744 (4)
*V* (Å^3^)	856.4 (3)
*Z*	4
Radiation type	Mo *K*α
μ (mm^−1^)	3.70
Crystal size (mm)	0.80 × 0.20 × 0.02

Data collection	
Diffractometer	Bruker APEXII CCD
Absorption correction	Multi-scan (*SADABS*; Krause *et al.*, 2015 ▸)
*T*_min_, *T*_max_	0.564, 0.747
No. of measured, independent and observed [*I* > 2σ(*I*)] reflections	23320, 3651, 3315
*R*_int_	0.049
(sin θ/λ)_max_ (Å^−1^)	0.809

Refinement	
*R*[*F* ^2^ > 2σ(*F* ^2^)], *wR*(*F* ^2^), *S*	0.030, 0.057, 1.11
No. of reflections	3651
No. of parameters	114
H-atom treatment	H atoms treated by a mixture of independent and constrained refinement
Δρ_max_, Δρ_min_ (e Å^−3^)	1.27, −1.92

Computer programs: *APEX2* and *SAINT* V8.40B (Bruker, 2016 ▸), *SHELXT2018/2* (Sheldrick, 2015*a* ▸), *SHELXL2018/3* (Sheldrick, 2015*b* ▸) and *OLEX2* (Dolomanov *et al.*, 2009 ▸).

## full crystallographic data

### Crystal data

**Table:**

C_8_H_7_IO_3_	*F*(000) = 528
*M**_r_* = 278.04	*D*_x_ = 2.156 Mg m^−^^3^
Monoclinic, *P*2_1_/*c*	Mo *K*α radiation, λ = 0.71073 Å
*a* = 4.3286 (8) Å	Cell parameters from 6811 reflections
*b* = 21.334 (4) Å	θ = 2.9–34.1°
*c* = 9.2941 (16) Å	µ = 3.70 mm^−^^1^
β = 93.744 (4)°	*T* = 90 K
*V* = 856.4 (3) Å^3^	Plate, pale yellow
*Z* = 4	0.80 × 0.20 × 0.02 mm

### Data collection

**Table:**

Bruker APEXII CCD diffractometer	3315 reflections with *I* > 2σ(*I*)
φ and ω scans	*R*_int_ = 0.049
Absorption correction: multi-scan (SADABS; Krause *et al.*, 2015)	θ_max_ = 35.1°, θ_min_ = 2.4°
*T*_min_ = 0.564, *T*_max_ = 0.747	*h* = −6→6
23320 measured reflections	*k* = −33→34
3651 independent reflections	*l* = −14→15

### Refinement

**Table:**

Refinement on *F*^2^	Primary atom site location: dual
Least-squares matrix: full	Hydrogen site location: mixed
*R*[*F*^2^ > 2σ(*F*^2^)] = 0.030	H atoms treated by a mixture of independent and constrained refinement
*wR*(*F*^2^) = 0.057	*w* = 1/[σ^2^(*F*_o_^2^) + 1.660*P*] where *P* = (*F*_o_^2^ + 2*F*_c_^2^)/3
*S* = 1.11	(Δ/σ)_max_ = 0.001
3651 reflections	Δρ_max_ = 1.27 e Å^−^^3^
114 parameters	Δρ_min_ = −1.92 e Å^−^^3^
0 restraints	

### Special details

**Table:**

Geometry. All esds (except the esd in the dihedral angle between two l.s. planes) are estimated using the full covariance matrix. The cell esds are taken into account individually in the estimation of esds in distances, angles and torsion angles; correlations between esds in cell parameters are only used when they are defined by crystal symmetry. An approximate (isotropic) treatment of cell esds is used for estimating esds involving l.s. planes.
Refinement. The O-bound H atom was located in a difference map and its position was freely refined. The C-bound H atoms were geometrically placed (C—H = 0.95–0.98 Å) and refined as riding atoms.

### Fractional atomic coordinates and isotropic or equivalent isotropic displacement parameters (Å^2^)

**Table:**

	*x*	*y*	*z*	*U*_iso_*/*U*_eq_	
I1	0.05964 (3)	0.25795 (2)	0.54095 (2)	0.01430 (4)	
O3	0.7859 (4)	0.39459 (9)	0.91310 (18)	0.0170 (3)	
O1	0.4603 (4)	0.56516 (8)	0.72915 (18)	0.0180 (3)	
O2	0.7790 (4)	0.51960 (9)	0.89865 (19)	0.0212 (4)	
C6	0.2313 (5)	0.34266 (10)	0.6295 (2)	0.0127 (4)	
C7	0.4516 (5)	0.34172 (10)	0.7453 (2)	0.0128 (4)	
H7	0.522507	0.302965	0.785568	0.015*	
C8	0.5675 (5)	0.39821 (10)	0.8018 (2)	0.0121 (3)	
C1	0.5827 (6)	0.62513 (11)	0.7783 (3)	0.0203 (5)	
H1A	0.499780	0.658300	0.713823	0.030*	
H1B	0.809060	0.624553	0.777897	0.030*	
H1C	0.522278	0.633151	0.876394	0.030*	
C5	0.1189 (5)	0.39860 (11)	0.5688 (2)	0.0161 (4)	
H5	−0.033554	0.398522	0.490504	0.019*	
C3	0.4577 (5)	0.45534 (10)	0.7425 (2)	0.0123 (4)	
C2	0.5823 (5)	0.51534 (11)	0.7990 (2)	0.0144 (4)	
C4	0.2359 (5)	0.45406 (10)	0.6259 (2)	0.0156 (4)	
H4	0.163744	0.492573	0.584857	0.019*	
H3	0.836 (8)	0.4252 (17)	0.929 (4)	0.025 (9)*	

### Atomic displacement parameters (Å^2^)

**Table:**

	*U*^11^	*U*^22^	*U*^33^	*U*^12^	*U*^13^	*U*^23^
I1	0.01408 (6)	0.01099 (7)	0.01764 (7)	−0.00031 (5)	−0.00056 (4)	−0.00134 (5)
O3	0.0173 (8)	0.0173 (8)	0.0153 (7)	0.0008 (6)	−0.0061 (6)	−0.0004 (6)
O1	0.0241 (8)	0.0104 (7)	0.0188 (8)	−0.0031 (6)	−0.0048 (6)	0.0007 (6)
O2	0.0210 (8)	0.0204 (9)	0.0211 (8)	−0.0015 (6)	−0.0083 (6)	−0.0031 (6)
C6	0.0114 (8)	0.0128 (9)	0.0138 (9)	−0.0012 (7)	0.0009 (7)	−0.0017 (7)
C7	0.0128 (9)	0.0120 (9)	0.0136 (9)	0.0008 (7)	0.0011 (7)	0.0006 (7)
C8	0.0103 (8)	0.0155 (9)	0.0105 (8)	0.0020 (7)	0.0003 (6)	0.0010 (7)
C1	0.0282 (12)	0.0114 (10)	0.0211 (11)	−0.0062 (8)	0.0005 (9)	−0.0021 (8)
C5	0.0178 (10)	0.0137 (10)	0.0158 (9)	−0.0004 (7)	−0.0061 (7)	0.0005 (7)
C3	0.0132 (9)	0.0108 (9)	0.0128 (9)	0.0003 (7)	−0.0010 (7)	0.0004 (7)
C2	0.0140 (9)	0.0152 (10)	0.0138 (9)	−0.0006 (7)	0.0007 (7)	−0.0011 (7)
C4	0.0179 (10)	0.0104 (9)	0.0177 (10)	0.0002 (7)	−0.0053 (8)	0.0012 (7)

### Geometric parameters (Å, º)

**Table:**

I1—C6	2.101 (2)	C8—C3	1.407 (3)
O3—C8	1.357 (3)	C1—H1A	0.9800
O3—H3	0.70 (4)	C1—H1B	0.9800
O1—C1	1.447 (3)	C1—H1C	0.9800
O1—C2	1.336 (3)	C5—H5	0.9500
O2—C2	1.220 (3)	C5—C4	1.379 (3)
C6—C7	1.391 (3)	C3—C2	1.472 (3)
C6—C5	1.394 (3)	C3—C4	1.400 (3)
C7—H7	0.9500	C4—H4	0.9500
C7—C8	1.395 (3)		
			
C8—O3—H3	107 (3)	H1A—C1—H1C	109.5
C2—O1—C1	115.15 (18)	H1B—C1—H1C	109.5
C7—C6—I1	119.85 (16)	C6—C5—H5	121.0
C7—C6—C5	121.9 (2)	C4—C5—C6	118.0 (2)
C5—C6—I1	118.20 (15)	C4—C5—H5	121.0
C6—C7—H7	120.3	C8—C3—C2	120.44 (19)
C6—C7—C8	119.4 (2)	C4—C3—C8	118.89 (19)
C8—C7—H7	120.3	C4—C3—C2	120.63 (19)
O3—C8—C7	116.94 (19)	O1—C2—C3	113.23 (18)
O3—C8—C3	123.3 (2)	O2—C2—O1	122.9 (2)
C7—C8—C3	119.80 (19)	O2—C2—C3	123.8 (2)
O1—C1—H1A	109.5	C5—C4—C3	122.0 (2)
O1—C1—H1B	109.5	C5—C4—H4	119.0
O1—C1—H1C	109.5	C3—C4—H4	119.0
H1A—C1—H1B	109.5		
			
I1—C6—C7—C8	178.99 (15)	C8—C3—C2—O1	178.4 (2)
I1—C6—C5—C4	−179.01 (17)	C8—C3—C2—O2	−1.0 (3)
O3—C8—C3—C2	1.0 (3)	C8—C3—C4—C5	0.9 (3)
O3—C8—C3—C4	178.8 (2)	C1—O1—C2—O2	1.2 (3)
C6—C7—C8—O3	−178.90 (19)	C1—O1—C2—C3	−178.26 (19)
C6—C7—C8—C3	0.9 (3)	C5—C6—C7—C8	−0.8 (3)
C6—C5—C4—C3	−0.9 (4)	C2—C3—C4—C5	178.8 (2)
C7—C6—C5—C4	0.8 (3)	C4—C3—C2—O1	0.6 (3)
C7—C8—C3—C2	−178.8 (2)	C4—C3—C2—O2	−178.8 (2)
C7—C8—C3—C4	−0.9 (3)		

### Hydrogen-bond geometry (Å, º)

**Table:**

*D*—H···*A*	*D*—H	H···*A*	*D*···*A*	*D*—H···*A*
O3—H3···O2	0.70 (4)	2.05 (4)	2.670 (3)	149 (4)
O3—H3···O2^i^	0.70 (4)	2.53 (4)	3.087 (2)	139 (4)

Symmetry code: (i) −*x*+2, −*y*+1, −*z*+2.
